# Editorial: The Microenvironment in Pancreatic Cancer and Therapeutic Strategies Targeting Microenvironment

**DOI:** 10.3389/fonc.2022.923982

**Published:** 2022-06-23

**Authors:** Wanxing Duan, Zheng Wang, Weikun Qian, Dan Qi, Qingyong Ma, Erxi Wu

**Affiliations:** ^1^ Department of Hepatobiliary Surgery, The First Affiliated Hospital of Xi’an Jiaotong University, Xi’an, China; ^2^ Department of Neurosurgery and Neuroscience Institute, Baylor Scott & White Health, Temple, TX, United States; ^3^ Department of Oncology, LIVESTRONG Cancer Institutes, Dell Medical School, The University of Texas at Austin, Austin, TX, United States; ^4^ Department of Surgery, Texas A&M University College of Medicine, Temple, TX, United States; ^5^ Department of Pharmaceutical Sciences, Texas A&M University College of Pharmacy, College Station, TX, United States

**Keywords:** pancreatic cancer, tumor microenvironment, desmoplastic reaction, immunosuppressive, radio-chemotherapy resistance

Recently, research on the tumor microenvironment (TME) of pancreatic ductal adenocarcinoma (PDAC) has become a research hotspot worldwide (Wu et al.). Previously, it was thought that a physical barrier formed by dense desmoplastic stroma around the tumor cells restricted the development of tumors. Currently, increasing evidence indicates that the remodeled stroma-rich and immunosuppressive TME in PDAC promotes tumor formation and progression by modulating the biology of tumor cells. Thus, targeting various stromal components and pathways in TME may serve as a promising strategy for PDAC treatment. This hot Research Topic introduces microenvironment in pancreatic cancer and therapeutic strategies targeting it.

## Emerging Trends and Research Foci in the TME of Pancreatic Cancer

Nowadays, the pathogenesis of PDAC and its treatment strategies have been a hotspot of pancreas research. Wu et al. did an interesting work by analyzing the literature related to the TME of PDAC over the past decade worldwide. Clustering analysis revealed that current research hotspots mainly focused on energy metabolism in hypoxic TME, cancer associated fibroblasts (CAFs) in regulating TME, accurate diagnosis, drug delivery, and potential new treatments. It is believed that targeting and remodeling the TME using a multimodal therapeutic strategy is promising for PDAC treatment.

## Targeting the Kinesin Superfamily of Pancreatic Cancer

Recently, accumulating evidence revealed that kinesin’s aberrant expression is involved in the development and progression of multiple cancers (1–3). Yang et al. systematically investigated prognostic values, genetic variations, and the tumor-promoting potential of kinesin superfamily proteins (KIFs) in PDAC by using bioinformatics analyses. The study (Yang et al.) revealed that 13 prognosis-associated KIFs were correlated with tumor stage, immune infiltration, cell growth, and mutation status of *KRAS* and *TP53*. Two genes *KIF20B* and *KIF21B* were used to construct a prognostic model based on each patient’s risk score. Considering the function of *KIF20B* and *KIF21B* in immune infiltration and tumorigenic, further in-depth mechanistic studies of them and clinical cohort validation should be explored in future research.

## Targeting Fibrosis and Extracellular Matrix in the Microenvironment of Pancreatic Cancer

Perez et al. reviewed the composition and the important role of the ECM in PDAC progression. In recent years, the strategies for targeting the ECM as an adjunct to chemotherapy in PDAC have made some progression. Li et al. have shown that degradation of hyaluronan (HA) by PEGylated recombinant human hyaluronidase (PEGPH20) partially reverses malignant phenotype and leads to depletion of tumor-associated matrix, creating an amenable environment for intra-tumoral drug delivery (4). Unfortunately, the Randomized Phase III Trial by Van et al. reported that some patients experienced strong adverse reactions to PEGPH20 and those who tolerated it did not have an improvement in overall survival (5). A previous study (6). showed that treating mice with Shh inhibitors in addition to chemotherapy improved tumor micro-vascular density and survival. Due to the heterogeneity of CAFs, a recent study (7). suggested that blocking the Shh signaling pathway may shift the stroma CAF populations from myofibroblast-CAFs (myCAFs) to inflammatory CAFs (iCAFs) and promote an immunosuppressive TME, which therefore limits the application of Shh inhibitors in PDAC treatment. Thus, more understanding of the fundamental cell biology is needed to identify the vulnerabilities in TME for developing new treatments.

## Targeting the Immune Microenvironment of Pancreatic Cancer by Using Special Histopathological Analysis

The immune-associated TME of PDAC is extremely complex, revealing its pathological relationship is important for predicting prognosis and treatment response of pancreatic cancer (8, 9). Large-section histopathology (LSH) has certain advantages over classic small-section histopathology (SSH) when they are applied to the pathological analysis of immune microenvironment. Ding et al. revealed that LSH effectively reflects the original tumor status and can effectively predict the prognosis of PDAC patients by analyzing clinicopathological parameters and 10 immune cell parameters. Studies have shown that there are fewer CD4 and CD8 positive cells in the immune microenvironment of pancreatic tumors, whose content is important for immunotherapy, and the content of NK cells, B cells and other cell markers can also predict the difference of immune microenvironment (10).

## Targeting the Microbial Microenvironment of Pancreatic Cancer by Using RNA-seq

Pancreas, previously thought to be a sterile organ, contains a lot of microbes (11). Many studies have detected the association between microbiome and cancer in an indirect way, which utilized samples from stool, oral, and saliva (12–14). Here, Yu et al. investigated four RNA-seq datasets with 582 PDAC tissue samples from four irrelevant studies. The authors found that several genera were distributed in PDAC tissue. Specifically, eight core microbiotas (*Kocuria*, *Streptococcus*, *Bacillus*, *Lactobacillus*, *Ralstonia*, *Staphylococcus*, *Acinetobacter*, and *Pseudomonas*) were identified to be prevalent and abundant in and across the four datasets. Moreover, sampling sites and tissue source had significant effects on microbial composition. Reasonable utilization of microbial composition may have important implications for the prevention and treatment of PDAC (15).

## Targeting the Neural Microenvironment of Pancreatic Cancer

Qin et al. described that honokiol suppresses perineural invasion of pancreatic cancer by inhibiting SMAD2/3 signaling. Cancer neuroscience has become an emerging discipline to research the interaction between the nervous system and cancer (16). Cancer cells can secrete neurotrophins to promote axonogenesis by increasing adrenergic or cholinergic signaling (17), also leading to an increased nerve density in the TME. Nerves in the TME can modulate cancer initiation, progression, or metastasis by modulating tumor-related signaling. Nerves can transport nutrients such as serine and glycine from nutrient-rich area to nutrient-low area, and neurons can release amino acids *via* their axons to metabolically support PDAC cell growth and survival under nutrient-limiting conditions (18). Perineural invasion reprograms the immune microenvironment by the crosstalk between pancreatic cancer cell and cholinergic signaling and formatting the immunosuppressive microenvironment (19). Thus, neural regulation of tumors and their TME can be regarded one of the novel actionable hallmarks of cancer.

## Perspectives

The overarching goal of this Research Topic was to highlight the scientific community with some recent advances made on the discovery of TME’s roles in pancreatic cancer and new therapeutic strategies targeting TME. Understanding of PDAC tumor progression and its TME as a tumor fate determinant, as well as the reciprocal tumor-TME interactions, will help us improve clinical outcomes. We believe that the concept of considering tumor and TME as the co-organizer is significantly important in current PDAC research in order to develop novel therapeutic targets and regimens for PDAC.

## Author Contributions

All authors contributed to the article and approved the submitted version.

## Funding

This work was funded by grants from Corbett Estate Fund for Cancer Research (62285-531021-41800, 62285-531021-51800, 62285-531021-61800, and 62285-531021-71800).

## Conflict of Interest

The authors declare that the research was conducted in the absence of any commercial or financial relationships that could be construed as a potential conflict of interest.

## Publisher’s Note

All claims expressed in this article are solely those of the authors and do not necessarily represent those of their affiliated organizations, or those of the publisher, the editors and the reviewers. Any product that may be evaluated in this article, or claim that may be made by its manufacturer, is not guaranteed or endorsed by the publisher.
